# Financialisation reinforced: the dual legacy of the covid pandemic

**DOI:** 10.1007/s43253-021-00053-4

**Published:** 2021-10-14

**Authors:** Photis Lysandrou, Taimaz Ranjbaran

**Affiliations:** 1 City University Political Economy Research Centre (CITYPERC), Department of International Politics, City University of London, London, UK; 2 City University of London, London, UK

**Keywords:** Financialisation, Covid pandemic, Colonisation of the future, Core-periphery divide, Global wealth tax, G20, G23

## Abstract

This paper examines the impact of the covid pandemic on the financialisation process, here viewed as the growing domination of the world’s bond and equity markets over the world’s product markets. Two major arguments are advanced. The first is that the pandemic has reinforced the functionality of financial market scale, which is that its continuing growth signifies nothing other than that government and corporate organisations are colonising the future to cope with the rising financial pressures of the present. The second argument is that the pandemic has also accentuated one of the more notable dysfunctional aspects of the continuing growth of financial market scale, which is its enforcement of a core-periphery divide between the advanced and emerging market economies that occupy the global financial system. The paper concludes with some policy implications of the analysis that includes the call for a global wealth tax.

## Introduction

This paper examines the impact of the covid pandemic on the financialisation process viewed here as the growing domination of the world’s bond and equity markets over the world’s product markets. The majority opinion amongst heterodox economists is that this growing domination is an anomaly, a deviation from the usual norms of economic development. As one commentator put it: “According to most streams of heterodox economics, the process of financialization is mainly a pathological process of evolution within capitalism that requires that capitalism be radically reformed or superseded” (Vercelli 2013, p. 41). The alternative minority opinion is that the continuing scale growth of the securities markets is an entirely normal development within capitalism in that it signifies governments’ and corporations’ attempts to find cost-efficient means of coping with the increasing financial pressures they face. One of these means is increased reliance on the securities markets, the rationale being that while large amounts of funds can be raised at the time of security issuance the repayments of these funds can be spread over intermittent points in the future. This simple observation has given cause for the claim that financialisation from a scale perspective manifests the colonisation of the future as a space that can take the overspill of the financial pressures of the present (Lysandrou 2016; 2021). The first of the two major arguments advanced in this paper is that governments’ experience of the covid pandemic supports this latter interpretation of financialisation in that it is difficult to see how their huge issuance of different-dated bonds to finance the costs incurred by the pandemic can be interpreted in any other way.

Alongside the functional aspects of the continuing growth of financial market scale, there are also several dysfunctional aspects, one of the most notable being the enforcement of a core-periphery divide in the global financial system. At a time when the world’s securities stocks dominate the world’s material output base, it is the fact that most of the world’s emerging market economies account for very small fractions of these stocks that explain why they are kept pinned to the periphery of the financial system through the gravitational force exerted by the large securities stocks created in the world’s advanced market economies. The second major argument advanced in this paper is that the same efforts made by the world’s governments to cope with the economic fall-out of the covid pandemic also serve to further consolidate their countries’ respective positions on the opposing sides of the global core-periphery divide.

The structure of the paper is as follows. Section 2 discusses the scale and functionality of financialisation. Section 3 discusses financialisation’s enforcement of a core-periphery divide in the global financial system. Section 4 discusses the covid pandemic’s dual impact on the financialisaton process. Section 5 discusses some policy implications of the analysis, one of which is the necessity for a global wealth tax. Section 6 gives some conclusions.

## The scale and functionality of financialisation

Financialisation, put simply, “summarises a broad set of changes in the relation between the ‘financial’ and ‘real’ sector, which give greater weight to financial actors or motives” (Stockhammer 2012 p. 121).^1^ Amongst the changes that have given ‘greater weight’ to the financial sector and have accordingly received much attention are those pertaining to its *scale (*world financial stocks now dominate the world’s material output base on which they rest), its *status* (from playing a largely peripheral role in domestic economies, the financial markets have moved to a more central position as attested by their growing influence on corporate priorities and on the policy actions of central banks and other official institutions), and to its *character* (from being largely passive, the financial markets have become far more active as attested by the large increases in daily trading volumes in many of these markets).^2^ The fact that financialisation has several different dimensions has prompted the conclusion that it “is not a standardised and linear process” (Lapavitsas and Soydan 2020). This conclusion is wrong in that while it may well apply to some dimensions of financialisation, it most certainly does not apply to that of its scale, the focus of attention in this paper. While world financial stocks amounted to about one and half times world GDP in 1980, they had grown to over three and half times world GDP by 2020, with the main growth driving factors being the expansion of the world’s securities markets. Thus, where in 1980 the combined world bond and equity stocks amounted to about $11 trillion, which was on a par with nominal world GDP for that year, by 2020 total bond and equity volumes outstanding amounted to $106.2 trillion and $97 trillion respectively as against a world GDP of $87.8 trillion (SIFMA 2021).

The principal security issuers are corporations, banks and governments. Business corporations are the dominant suppliers of equity with banks in second place, while this ordering is reversed in the bond markets where banks are the leading bond issuers (‘financial bonds’), followed by governments and corporations. This bond issuance breakdown by institutional category indicates that there are two distinct sets of factors that are currently driving security market growth. The first set relates to the pressures of corporate production. In an era of rapid technological innovation and thus of ever intensifying market competition, business corporations must have constant access to large external sources of funds to finance research and product development, or to finance mergers and acquisitions, or to finance any of the other measures needed for survival. Corporations across all regions tend to rely on a mix of debt and equity forms of external finance to supplement their funding needs to avoid an excessive concentration of risk on the one hand and an excessive dilution of property ownership on the other. The major cross-regional difference concerns the composition of external corporate debt in that US corporations have a strong preference for bond-based borrowing as opposed to bank-loan borrowing while the reverse is true of European and Japanese corporations. The second set of factors that is driving security market growth relates to a wider array of socio-economic pressures, the most significant being those associated with demographic change. Commercial banks have traditionally relied on household deposits to fund their loans to businesses and households, but the fact that households are now on average living far longer after retirement than was previously usual means that many of them are shifting their retirement savings out of bank deposits and into financial market investments in the search for higher yield. As a result of this change in household savings behaviour banks are having to considerably increase their issuance of long-term bonds and short-term money market instruments to fill the gaps in the liability side of their balance sheets. Finally, the pressure of demographic change^3^ is also the principal factor behind the rise in government borrowing, as governments, faced with rising pension and healthcare costs in addition to a range of other spending commitments, have had to increasingly rely on bond issuance to help finance these costs.^4^

The upshot of the above observations is that recent decades have seen a radical change in borrowing organisations’ dependence on the bond markets. Previously, that dependence tended to be transitory as corporations or governments resorted to bond issuance to finance a particular large-scale project or to help defray the costs incurred by a particular emergency. This tendency was consonant with the idea of the closed monetary circuit familiar from bank-based financing of corporate production: the circuit is opened when funds are borrowed from lenders and is then closed when the funds are returned upon completion of the investment project. What has now changed is that corporate and government dependence on the bond markets has become permanent as a direct result of the permanence of the new types of financial pressures bearing down on them. The consequence of this development is that the typical debt issuance-debt redemption relation is no longer one that is representative of a single closed circuit spanning a fixed period, but rather one that is part of an open-ended series of circuits that stretch across indefinite spans of time as bonds that reach their maturity dates are replaced with new bonds and so on in a continuum. There is, of course, a precondition for this constant bond rollover to be possible, which is that there must be a body of lenders on the demand side of the bond markets whose investment requirements oblige them to also hold large amounts of bonds on a permanent basis. The reality is that such a body does now exist courtesy of the same underlying socio-economic pressures that have helped cause a rise in permanent bond borrowing in the first place.

While there are several other agents who are important buyers of financial securities, including very wealthy individuals, central banks, sovereign wealth funds and hedge funds, by far the most important group of buyers when taken collectively are the institutional asset managers, the pension and mutual funds and insurance companies. For long, a small cottage industry catering for the very wealthy, asset management has become in many countries a mass industry catering for retirement and other welfare arrangements of large sections of the population. With this growth in asset management scale has come a corresponding growth in the need for investable assets, and most notably for equities and bonds. Although there are other types of assets that can serve as stores of value, financial securities necessarily comprise the majority proportion of institutional asset holdings because what sets them apart from other asset classes is their potential ability to combine a yield generating property with the properties of liquidity (they can be converted into cash with minimal impact on price) and tradability (they can be circulated without restriction amongst investors).^5^ Asset managers also hold bank deposits for liquidity purposes but to a limited extent because while they have high liquidity, they have low yield, and they also hold real estate assets but again to a limited extent because while these can generate relatively high yields, they have low liquidity. Rather, the exigencies of asset managers’ role as financial intermediaries that market asset portfolios to the public require them to hold the bulk of these portfolios in the form of liquid financial securities, assets into which clients’ monies can be easily poured and from which monies can be easily extracted to pay clients. A large volume of demand for corporate and government securities is thus ensured, but what is also to the point is that this volume demand will remain permanent given that the economic costs of population ageing will likely force governments to increasingly shift away from universal welfare provision towards more selective forms, thus forcing increasing numbers of households to enlist the services of asset managers when making their own private welfare arrangements.

The pressures of asset management that require pension and mutual funds to hold the bulk of their asset portfolios in the form of corporate and government securities are also those that require them to impose certain tight preconditions on these security issuing organisations. As equities and bonds have no intrinsic value, simply being claims on future income streams, it follows that their quantitative value storage capacity depends entirely on guarantees that cash is returned to investors at an agreed rate and at agreed time intervals. To ensure these guarantees, security issuing organisations must comply with two sets of behavioural standards, governance standards in addition to production and service provision standards. Compliance with production standards is necessary for the obvious reason that without some demonstrable commitment to them on the part of security-issuing organisations there can be no reasonable guarantee of the size and stability of the income flows against which claims are made. While necessary to the value storage function of securities, however, this first compliance is not sufficient. Corporations can excel in production but decide not to distribute cash to investors for one reason or other. Similarly, governments can excel in service provision and generate tax revenues accordingly but still give a low priority to the payment of interest on bonds. For sufficiency, a second set of behavioural standards is required, governance standards. Broadly defined, the governance of an organisation concerns the way in which it conducts its affairs to meet the different priorities of its various stakeholders. From the standpoint of investors, the question of corporate or public sector governance boils down to the level of priority given to their interests as shareholders or bondholders: high priority means that there is a reasonably good guarantee that cash will be returned to them in the required amounts and at the required intervals, whereas a low priority means that there is little guarantee that cash will be returned.

The fact that most financial securities in circulation today have acquired solidity as determinate quantities of value by virtue of the governance standards that run in parallel with production standards is key to the growing scale disparity between financial securities stocks and annual material output flows. As noted at the outset of this paper, for many heterodox economists, this disparity is an anomaly. Indeed, no other aspect of financialisaton is so problematic and vexing to heterodox economists than that of financial scale. They accept that the financial sector must reach a minimum size to effectively serve the productive and allocative needs of the real sector, but they consider its current size to be far in excess of that minimum.^6^ There is simply too much finance (Lapavitsas 2010; Seccareccia 2012; Lavoie 2012; Epstein 2013). The financial sector, as Epstein and Crotty (2013) put it, is simply “too big”. The assumption behind this standpoint is that financial scale should ultimately be determined by just one set of drivers, those relating to production, in which case the growth rate of the world’s securities markets should be in line with that of world GDP. On the contrary, the fact that the growth rate of the securities markets now consistently outstrips that of world GDP would appear to indicate that there is a second, non-production related set of financial scale drivers that, by dint of being non-production-related, must be speculative, or in some other way dysfunctional, in purpose and content.^7^ Our position is that there are financial scale drivers that, while having no direct relation to the needs of production, are nevertheless functionally necessary in that they relate to the financial pressures of ongoing socio-economic change. Put the production-related and socio-economic related drivers together and it becomes clear that the continuing expansion in financial market scale serves the real economy in that it serves the vital interests of the organisations that are the major producers and service providers in that economy. Just as corporations and governments on the one side need to continually roll over their debt securities to cope with their ever-increasing financial commitments, so must institutional asset managers on the other side need to continually buy those securities to meet their own ever-increasing financial commitments. The colonisation of the future is indeed an apt way of describing ongoing financial market growth because the future is indeed being harnessed as an adjunct space where governments and corporations can permanently store their different dated liabilities and where asset managers can serve as the permanent custodians of these liabilities. The behavioural standards necessary to the solidification of securities’ value storage capacities in the end ties in with this colonisation of the future. As the future is unknown, uncertainty can never be eliminated, but what the various behavioural rules and benchmarks now used to monitor and control the risks on financial securities do is to make uncertainty sufficiently manageable as to make the future sufficiently fit for permanent occupation.

## Financialisation’s reinforcement of a core-periphery divide

To recognise the functionality of the continuing scale growth of finance is not to ignore the fact that this same development can have dysfunctional consequences. One of the most significant of these is the unbalanced structure of the global market-based system. Following the collapse of communism in the 1990s, virtually all the world’s two hundred or so independent nation states now occupy this system and operate to its common standards and rules of engagement. However, financialisation ensures that they do so on a highly unequal basis, with a minority constituting the core of the system and the great majority pinned to its periphery. A core-periphery divide in the international economic domain is not something new, but what is new is the pivotal role played by the highly uneven geographical breakdown of the world’s financial markets in perpetuating such a divide.

As previously noted, world total bond and equity volumes outstanding at end-2020 were $106.2 trillion and $97 trillion respectively. Of the advanced market economies, the US’ average share of these combined volumes was 40% as compared with the EU 27’s 15% share, Japan and the UK’s respective shares of 6.3% and 3.4% and the 8.5% share of the other advanced market economies (AMEs). Of the emerging market economies (EMEs), China’s average share of the combined bond and equity volumes was 13% while all the other EMEs together accounted for the remaining average of 6% (SIFMA 2021). These differences in the regional contribution to world securities stocks translate into even more significant size differences between regions when these stocks are broken down according to currency of denomination. From this standpoint, the only currency areas that host enough securities as to give them a substantive presence in the global financial landscape alongside the US dollar are the euro, the yen, the pound sterling and the renminbi. All the other currency areas as defined in security market terms reduce to fragments, a fact that finds reflection in their extremely small percentage shares of the $6.6 trillion daily turnover in the foreign exchange markets (thus where at one extreme the dollar currently accounts for about 44% of this turnover, at the other extreme the largest of the EMEs register between 0.5 and 2% while all the smaller EMEs register barely perceptible percentage ratios, BIS 2019). In a hypothetical world of no international capital mobility, these regional size differences as measured by securities stocks would remain mutually independent in that they would be determined solely by factors that are internal to each region. By contrast, in the current real world of high international capital mobility dominated by foreign portfolio investments (as distinct from foreign direct investments)^8^ regional security market size differences are mutually interdependent, and they are so in large part because of the uniform standards used to quantify the different types of risks that are priced into securities.

Recall that as securities have no intrinsic value, portfolio investors need to hold security issuing organisations to various behavioural standards to ensure the reliability of cash returns and hence the tangibility of securities’ quantitative value storage capacities. A further point is that if portfolio investors are to be able to choose between which securities to hold and to decide in what quantities they need to be able to compare the risks on different securities. Given that national governments have the power of taxation, their bonds typically serve at the domestic level as the risk-free benchmarks against which corporate credit risk premiums are calculated and priced (as also are the corporate equity risk premiums). At the international level, there are two basic variations regarding risk calculations. One is that national government bonds are now themselves subject to comparison to internationally accepted risk benchmarks, typically represented by the bonds of the governments of the largest and most powerful market economies. The other is that currency exchange rate risk now appears alongside credit risk as the other major type of risk that also requires internationally sanctioned benchmarks for its quantification and pricing into tradable securities. As there is no single world currency, it is the national currencies that are most widely used in an international capacity that are taken as the currency risk benchmarks, which in practice means the US dollar followed by the euro. These uniform risk pricing standards may appear to be neutral in that in being sanctioned by all agents that are active in the global economy they do not belong to any one group of agents. The reality is very different. All individual securities form constituent parts of a particular aggregate mass of same currency denominated securities, and what this means is that the benefits accruing from the common use of international risk pricing standards are distributed in direct proportion to the size of the currency mass: the larger the mass, the greater the benefits and vice-versa.

To illustrate the point, let us first consider the US securities markets. These are by far the largest and deepest in the world, and as such they are highly attractive to foreign investors in that not only is there an abundance of securities in which to store their funds, but also a wide choice range of different securities across which they can move funds according to economic circumstances. However, these advantages to foreign investors must be paid for in the sense that they will on average earn comparatively low returns on their dollar assets. Thus, for example, they will earn no currency risk premium (due to the range of choice of US asset classes across which investments can be moved according to any change in economic conditions without being subject to exchange rate frictions); low credit risk premiums (due to the general strength, reputation and uniformity of the US legal and governance infrastructure); low liquidity risk premiums (due to the depth of the US securities markets and hence the ease of trading with minimal price impact); and a low sovereign risk premium (due to the scale of US domestic economic activity and hence the corresponding government power of taxation). By contrast, foreign investors can on average earn comparatively high returns on investments in countries that host small local currency-denominated securities markets because in these cases there will be currency risk premiums (any cross-security flows generated by any change in economic conditions will also typically take on a cross-currency dimension), high credit risk premiums (that may reflect a weak legal and governance infrastructure as much as a small domestic production base for local corporations), and high sovereign risk premiums (reflective of small domestic tax bases whose smallness may again be the result of a small domestic economy as also of a weak legal infrastructure). It reflects how wide the current divergence between country risk premiums is that the aggregate annual returns to foreign investors from their dollar assets are consistently lower than the annual returns to US investors from their foreign assets even while the aggregate amounts of foreign investments in the US are consistently higher than the amounts of overseas US investments (Forbes 2010; Piketty 2014).^9^

It is here that we can see why national capital market sizes are mutually interdependent in a world of international capital mobility. We have said that foreign portfolio investors are drawn in large numbers to the US equity and bond markets because these are the world’s largest and deepest, but in being so drawn to these markets they then contribute to their further growth in size and depth, thus contributing to the ease with which the US’ government and corporations can issue substantially more securities before coming up against interest service constraints. Thus, the large mass of dollar securities continually begets an even greater mass as foreign investors are continually willing to trade comparatively low returns on their dollar assets off against the various benefits accruing from these assets thereby enabling the US government and US corporations to continually issue increasing amounts of securities on an affordable, cost-effective basis. By contrast, a small mass of local currency denominated securities produced by a country perpetuates continuing smallness as foreign portfolio investors in these securities demand such high returns as compensation for the various risks attaching to them as will seriously constrain the amounts of securities that can be safely issued by the country's organisations.

In sum, the new form of enforcement of a core-periphery divide in the contemporary global economy is the direct result of the fact that financialisation from a scale perspective is as much a *standardised* process as a *linear* one. If it is the interplay between the financial commitments of government and corporate borrowers on the one side and those of the institutional investor lenders on the other that is driving the growth of financial security volumes at an increasing rate, it is the use of common standards for monitoring and controlling the risks on securities that, by solidifying the latter’s value storage capacities, gives mass to a given aggregation of same-currency denominated securities and a corresponding power of attraction that varies in direct proportion to the size and density of the currency mass. It is this power of attraction that marks off the contemporary era from all previous historical eras. Previously, it was the exercise of conscious control on the part of agents and institutions in the core countries that was the central means by which other countries and their agents and institutions were kept in a subordinate position. This control may have taken on overt and blunt forms as in the colonial era, or more covert and subtle forms as in the immediate post-second world war era, but the common denominator was that the control used to maintain a core-periphery divide in the international arena was based on acts of conscious and deliberate intention. Such acts still feature today but no longer as the typically representative means by which most countries are kept pinned to the periphery of the international economic system. The agents and institutions of the countries that host large securities markets can make all their decisions and frame all their actions and policies solely with reference to their own internal interests and priorities and the countries that host small securities markets will remain pinned to the periphery by virtue of the gravitational force exerted by the large markets.

## The covid pandemic’s dual impact on financialisation

In turning to the covid pandemic’s implications for the scale dimension of financialisation, we first recall three observations: that the largest of all the financial markets in the contemporary era are the bond markets; that governments continue to be amongst the major suppliers of bonds because their dependence on the bond markets is now both significant and permanent; and that this permanent dependence is due not only to production-related factors (such as government investments in physical infrastructure or government aids to industry) but also, and in many national cases more importantly, to a wider array of socio-economic related factors, including those associated with ongoing demographic change. The financing costs of dealing with the devastating effects of the covid pandemic essentially represent yet a further addition to the list of socio-economic drivers of bond market scale.

To begin with, the covid crisis has placed government finances under strains that have been ever heavier than was the case following the financial crisis of 2007–2008 for reasons that include the fact that the economic costs of the covid pandemic came on top of the health care costs that had to be borne by governments, the fact that it was not just one group of firms in just one economic sector that had to be protected from bankruptcy by government bailout loans but a whole range of firms drawn from across the entire domestic economy, and the fact that on this occasion governments had to fund not only business bailouts but also the wages of employees that had been temporarily laid off while also the bearing the financial costs of the soaring levels of unemployment. The inevitable result of these multiple strains on government finances was a sharp upward spike in the rate of government bond issuance. Thus, in just the first five months of 2020, OECD governments issued about $11 trillion worth of bonds, an amount almost 70% higher than the average amount issued in the same period over the previous five years. Netting out bond redemptions, outstanding central government debt for the OECD area increased from $47 trillion in 2019 to $52.7 trillion by the end of 2020, a figure that was $3.5 trillion higher than the pre-COVID estimate.^10^ A further point worthy of note is that during 2020, more than two-thirds of OECD governments opted for an increased issuance of government securities across the yield curve and introduced new maturity lines. For example, Germany’s government added 7- and 15-year maturity bonds to its borrowing programme in April, 2020, while the French and US governments launched new 20-year bonds in May, 2020, and the Italian and Spanish governments launched new 50-year bonds in February and April, 2021, respectively.

For governments to be able to issue such vast amounts of bonds across the maturity spectrum, there must be a body of institutions with a corresponding absorption capacity. Central banks obviously play a vital role in this respect. In normal times, these act as the lenders of last resort to national governments in that they stand ready to buy whatever amounts of marketable government bonds that are not taken up by the private sector.^11^ In times of crisis, this backstop role increases in importance as was witnessed during the financial crisis and during the subsequent covid pandemic crisis. This said, it is still the case that a further substantial proportion of government bonds are held by an array of institutional investors, most notably by the pension funds and mutual funds and insurance companies. We have already observed that in promoting the transformation of asset management into a mass industry through forcing higher income households to make their own welfare arrangements, governments have thereby also helped to create a sizable and permanent base of demand for their bonds. The further observation to add here is that the size and permanence of this demand base is due to the important role played by government bonds as the operational core of institutionally managed bond portfolios. At all times, asset managers hold substantial amounts of government bonds in their bond portfolios not only on account of their greater liquidity and information-insensitivity but also on account of their greater safety as stores of value. What differ at different times are the exact amounts of government bonds held as the safe-haven core of bond portfolios in that this core tends to contract at times of economic prosperity when institutional investors feel confident enough to allocate more funds to higher yielding corporate securities, and to expand at times of economic downturn when these investors place a greater premium on safety than on yield.^12^ We saw this phase of expansion play out during the financial crisis and we saw it play out again during the covid crisis, only this time to an even higher degree as asset managers sought the safety of government bonds on a level rarely seen before in their short history as a mass industry.

An important caveat to the above is that when speaking of the institutional investor flight to the safety of government bonds in times of economic crisis, it is the bonds of the governments of advanced market economies that are sought, not the bonds of the governments of the emerging market economies. Virtually all of these economies have been hit by the covid pandemic to an extent that has been far greater than was the case following the financial crisis, because on that occasion, they continued to function relatively normally even while there was a temporary contraction of their overseas markets. With the covid crisis, however, things have been very different for the EMEs because on top of the huge disruption to their domestic production and employment levels caused by the pandemic, and on top of the sharp falls in their export earnings caused by the contraction of their overseas markets, they have also had to face falls in the price of oil and in other commodity prices, falls in remittances from abroad and last, but not least, falls in incomes from a range of tourism and other travel-related services. As in the case of AME governments, EME governments have had to step up efforts to protect their economies from complete collapse and, as in the case of the former, the latter have had to resort to increased bond issuance to finance those efforts. This, however, is where the similarities stop because the terms under which EME governments have financed their economic rescue efforts have been as onerous as have been favourable the terms under which AME governments have financed their rescue efforts.

At the beginning of March, 2020, the FTSE World Index of 10-year government bond yields stood at around 2% and by the beginning of February 2021, it had risen to just over 3% (ICMA 2021). All through that year the average yield on 10-year government bonds issued by OECD member states was about 1%. Even before the covid crisis, the yields on OECD government bonds had already trended down in the years following the financial crisis due to the dampening effect caused by institutional investor flight to safety. Thus, according to OECD estimates, where the average 10-year government bond yield was around 5% in 2006, that figure had fallen gradually to around 1.5% in 2019 and then to 1% by March 2020 despite the surge in OECD government debt issuance.^13^ By contrast, there was a greater upward-level dispersion of the yields on the EME 10-year government bonds over this latter period, the range stretching from an average of 3.2% on China’s bonds to over 15% on the bonds of many of the smaller EMEs (UNCTAD 2020). It is testimony to how wide was the gap between AME and EME government bond yields all through 2020 and into the beginning of 2021, that the FTSE World Index could rise by 1% from 2 to 3% even while the aggregate amount of EME government bonds issued over this period was a small fraction of the world total amount of government bond issuance. A key contributory factor to these contrasting terms of government bond financing was the contrasting direction of portfolio investment flows. In the space of just one month, March, 2020, close to $1 trillion had been withdrawn from EME securities, initially from EME equities but then subsequently also from EME bonds (ICMA 2020). Several AMEs benefited from these outflows from the EMEs, but none more so than the USA. Faced with the exceptional severity of the covid pandemic’s damaging impact on its domestic economy, the US government had responded with an equally exceptional increase in treasury bond issuance. Thus, where total US treasuries amounted to $19.2 trillion in 2019, that figure had risen to $23.1 trillion in the first six months of 2020 alone (SIFMA 2020b). Despite the fact that this aggregate volume increase was particularly marked by heavy increases in the longer dated treasuries (that now included 20-year bonds), the yields on these not only did not go up but also continued to hover near zero rates, the steep rise in foreign demand for US treasuries being a major contribution to this development. Thus, where foreign central banks and private investors held $6.2 trillion worth of US treasuries in 2019, that figure had risen to $7.1 trillion by mid 2020 (SIFMA 2020b).

Having pulled out vast sums from the EMEs between March and early April, 2020, portfolio investors then began thereafter to direct funds back to the EMEs in the quest for high yields. The point was that if the world’s portfolio investors were sacrificing yield in favour of safety when keeping the core of their asset portfolios in the form of AME government and blue-chip corporate securities, they had to find yield elsewhere, and that elsewhere following the outbreak of the pandemic could only be EME government bonds. The inevitable result of the comparatively high yields that EME governments have had to pay out on their bonds was a severe restriction on the extent to which they have been able to implement economic recovery programmes. Following the outbreak of the covid pandemic and the extensive damage to the global economic architecture that it wrought (the estimates indicated that the virus reduced economic growth in 2020 to an annualised rate of between − 3.4% and − 7.6%, CRS 2021) the debate was whether there would be a V-shaped recovery or rather an L-shaped recovery, one marked by a prolonged slow and hesitant rate of growth punctuated by many gaps, uncertainties and possible reversals. However, as has been pointed out by economists based at UNCTAD (UNCTAD 2020), the more likely scenario will be a K-shaped recovery, a high recovery rate for the AMEs coupled with a low recovery rate for the majority of the world’s EMEs. This prediction will likely turn out to be correct, not only because so many of the EMEs were amongst those that experienced the highest rates of contraction in 2020 (World Bank 2020) but also because of the huge disparities in the costs of financing the post-covid recovery programmes.

The conclusion that falls out of the above discussion is that if the covid pandemic has given further impetus to the scale growth of the global financial markets by virtue of governments’ response to the pandemic, it has also by that same token served to further consolidate the core-periphery divide in the global financial system. For the governments of the advanced market economies, the relation between financialisation from a scale perspective and their efforts to counter the impact of the pandemic has been one of mutual facilitation: where the continuing heavy issuances of government bonds will give a continuing boost to financialisation going forward, it is because of financialisation’s past development, powered in large part by the interplay between governments and institutional asset managers, that governments have been able to contain borrowing repayment costs in the face of expanding borrowing volumes. The exact reverse situation has held for the governments of the emerging market economies: where financialisation’s past development has given a new dynamic to their countries’ peripheral status in the global economy, their efforts to contain the negative effects of the covid pandemic have only served to further affirm that peripheral status.

## Some policy implications

The most immediate implication of the foregoing discussion is that financialisaton from the perspective of continuing bond and equity market growth cannot be reversed. It might be thought that a policy aimed, say, at downsizing the bond markets in favour of a return to a primarily bank-based form of finance would be a desirable initiative in that the relational nature of this form is conducive to those productive investments that require that finance be provided in a way that gives protective shelter against financial market pressures. But, to again repeat the point, alongside the production-related factors behind the financing needs of large organisations, there are also various socio-economic related factors, and these factors entail a need not so much for *protection* against financial market pressures as for an *accommodation* of these pressures. Nothing better exemplifies this point than the correlation between the rising financing costs of demographic change and rising government bond volumes, a correlation that, having become firmly established in the decades since 1980, looks set to continue over the coming decades. If population ageing is the key driver of population growth in many of the world’s advanced market economies, it is the increasing birth rate that is the key driver in the world’s emerging market economies. Taken together, these population developments are placing huge pressures on the world’s natural resources, pressures that are in one way or another causing the climate change that is at the root of the many types of natural disasters now occurring with unprecedented frequency and that are, therefore, placing further financial burdens on national governments. Into this mix of socio-economic factors that are forcing governments into an ever-increasing dependence on the bond markets, come the costs of coping with the effects of recurring global pandemics. The covid-19 pandemic may have been the first global pandemic of the twenty-first century, but all the scientific predictions are that it will not be the last. On the contrary, ongoing population growth and its attendant environmental pressures, taken in conjunction with the closer physical interconnection of the world’s countries made possible by the rapid pace of change in transportation and communication technologies, ensure that the effects of any new virus that appears will have immediate global reach and impact.

To say that the continuing growth of the world’s financial markets cannot be reversed is not to say that there cannot or should not be any interference in that growth. On the contrary, the fact that the hugely uneven breakdown of world securities stocks by currency of denomination serves to give both new form and content to a core-periphery divide in the global economy gives cause for interventionist policies that can correct for the inequalities that result from that divide. Foremost amongst such policies must be a global wealth tax. Calls for such a tax to be placed on the policy agenda have been made before by several institutions (e.g. Tax Justice Network) and individuals (e.g. Piketty 2014; Lysandrou 2019) and it is possible that the experience of the covid pandemic will give added urgency to these calls as we shall see in a moment. First, we must note that where the interplay between the financing needs of corporations and governments and the financial commitments of institutional asset managers has been the driving force behind the ongoing scale growth of the world's securities markets, it is the very wealthy individuals — those classified as high net wealth individuals (HNWIs) — who have been amongst the major beneficiaries of this growth.

The original sources of private wealth creation are many and varied, but there are basically only five asset classes that subsequently serve in a wealth storage capacity, the two foremost being equities and bonds, followed by cash, real estate, and alternative investments (Capgemini 2021). It is the fact that the world’s HNWIs tend on average to hold about one half of their wealth in the form of financial securities, coupled with the fact that the annual growth rate of the world’s securities stocks consistently out-strips the annual growth rate of world GDP, that largely explains the continuing massive concentration of wealth in the hands of a vanishingly small percentage ratio of the world’s population. Furthermore, another contributory factor to private wealth accumulation is government reliance on monetary policy as the most favoured tool for macroeconomic management, a factor that has become particularly significant in periods of economic downturn and monetary loosening. Thus, for example, in the eight years between 2007 and 2015 during which time much of the world’s population suffered the effects of the great financial crisis and bore the brunt of austerity measures many of which had resulted from government-financed bank bail-outs, the world’s HNWI population had risen from 10 to 15 million while their combined wealth had risen from $41 trillion to about $59 trillion with much of this rise having been caused by the financial asset price inflation that was the inverse result of the sharp drop in interest rates due to quantitative easing (Capgemini 2016). The experience of the covid pandemic has provided an even more striking example of how far private wealth accumulation is parasitic on bond-financed macroeconomic policies. In 2020, the first full year in which the catastrophic effects of the pandemic played out with the result that the world’s economies contracted at rates of between − 3.4% and − 7.6%, aggregate wealth held by the world’s HNWIs had, in stark contrast, expanded by about 8%, from $74 trillion at end-2019 to $79.8 trillion by end-2020 (Capgemini 2021).

A necessary first step in any implementation of a global wealth tax is the establishment of a global tax authority that can serve as the central coordinating and monitoring body. As recent experience has shown, any national initiative for taxing wealthy individuals will simply not succeed given the ease with which these individuals can move both themselves and their wealth across borders. In addition to achieving the necessary degree of coordination, a further reason for the establishment of a global tax authority is that it could also serve in a distributive capacity, allocating the substantial funds raised from the very rich to the governments of the countries where those funds are most needed. From the foregoing analysis regarding the way that financialisation has given a new operational dynamic to the core-periphery divide in the world economy, it would follow that there are two overlapping criteria that must form the basis for fund allocation: namely, that in addition to the poverty criterion — the greatest amounts of funds should be allocated to the governments of the world’s poorest counties — there must also be the borrowing cost criterion — the greatest amounts of funds should be allocated to the governments of countries that face the highest interest charges in the world’s bond markets. It may be that part of the explanation as to why so many governments face high borrowing costs is domestic mismanagement or financial malpractice. However, even if abstraction is made from these factors, borrowing costs to governments of countries that host small domestic financial markets would still be higher than those costs to governments of countries that host large markets due to the corresponding variations in the amounts of the different types of risks that are priced into financial securities. Thus, in counterbalancing these risk-induced variations in governments’ borrowing costs, a policy of distributing funds to governments in inverse proportion to the size of their domestic financial markets would be entirely justified because this policy would be entirely fair.

Although the implementation of a global wealth tax will face many difficulties given the fierce resistance that such a policy will encounter, the astonishing degree to which wealth accumulation is concentrated at the very top of the private wealth pyramid will possibly make it easier to overcome these difficulties. Between end-2019 and end-2020 during which time world HNWI wealth grew by nearly $6 trillion from $74 to nearly $80 trillion, over one half of this amount went to the world’s ‘ultra-HNWIs’, individuals with net assets of over $30 million. Numbering just over 186,000 and holding 34% of the $74 trillion HNWI wealth (i.e. about $24.6 trillion) at end-2019, they numbered just over 200,000 and held 33.6% of the $79.9 trillion HNWI wealth (i.e. about $28 trillion) at end-2020 (Capgemini 2021). As over 90% of the ultra-HNWIs are domiciled in the world’s major advanced market economies (the USA on its own is home to about 30% of these individuals), in other words, in those economies that host the largest equity and bond markets, it is clear that the sole reason why so much private wealth could have accumulated so quickly in the hands of so few individuals at a time of global economic contraction was the inflationary impact on the prices of AME government and blue chip corporate securities caused by the combination of AME monetary policy loosening and the steep rise in the proportion of global portfolio investments seeking the safety of the AME financial markets. To the extent that all these disturbing facts and figures are widely advertised and made public, so should there be a corresponding rise in public anger and disgust, which could in turn be funnelled towards support for a global wealth tax to be levied in the first place on the world’s super-rich, the 200,000 or so ultra-HNWIs.

## Conclusion

There is little doubt that the covid pandemic’s long-term consequences for the global economy will be the subject of much analysis and debate. The focus of attention here has been on one of the pandemic’s more immediate consequences, the boost given to financial market scale. As sudden as it was massive, that boost served to further accelerate a scale growth of finance that had already been gathering momentum in recent years. In so doing, it re-affirmed the functionality of that scale growth while also re-affirming several of its dysfunctional off-shoots. As regards functionality, the view that the continuing expansion of the securities markets represents nothing other than the colonisation of the future has until now remained a minority view. Following the pandemic, however, it is possible that it will attract more widespread attention and support because it will be hard to interpret the issuance of huge amounts of government bonds of all maturities as anything other than governments’ use of the future as a space into which the swelling financial pressures of the present are released. As regards dysfunctionality, the substantial differences in governments’ abilities to finance their post-covid recovery programmes has exposed the extent to which the universal use of common standards for pricing financial securities not only makes possible the continuing scale growth of the financial markets but also serves to continually keep most of the world’s governments pinned to a peripheral position in those markets. As financial scale growth cannot be reversed because of its functionality, the only meaningful way of correcting for the negative effects of its enforcement of a core-periphery divide in the global financial system is to establish new global financial institutions that are equipped with the necessary powers of intervention and correction. This paper has suggested the creation of a global tax authority charged with implementing a global wealth tax as a first step in this direction.

## Footnotes

1 There are several variations of this definition of financialization, but the one that continues to be most frequently cited is that given by Epstein: “financialization means the increasing role of financial motives, financial markets, financial actors and financial institutions in the operation of the domestic and international economies.” (Epstein 2005, p. 3).

2 Fine (2011) and Sawyer (2013–2014) suggest that there are eight features of financialization, but these are essentially variations of the three key features involving size, status, and character of the financial sector.

3 As regards demography, what sets the advanced economy countries apart is the low rate of population growth combined with a high rate of population ageing (for example, the median age of the population in North America and West Europe rose from 32 in 1980 to 41 in 2010, while the median age in Africa over that same period only rose from 18 to 20. (United Nations 2019) This combination has led to a trend rise in old-age dependency ratios (defined as the number of individuals aged over 65 per 100 individuals aged between 20 and 64). Thus, according to recent OECD estimates, the dependency ratio across all OECD countries roughly doubled from 13.9 in 1950 to 27.9 by 2015 and is expected to reach 35.2 by 2025 (OECD 2019a).

4 Closely correlated with population ageing and the rise in the old age dependency ratios over recent decades has been the rise in government social spending as a percentage of GDP (from an average percentage share of just 8% in 1960, that share had risen to an average of 17% across the OECD countries by 1990 and to an average of 20% in 2018) and as a percentage share of total government expenditure (the average share for the EU-28 countries in 2018 was 40%, but closer to 50% for the UK and other northern European counties, a figure similar to that for the USA) with pensions and health care provision being the two largest components of government social spending. Ortiz-Ospina and Roser (2016); OECD (2019b), *Social Expenditure Update;* Kenworthy (2019)

5 According to a recent OECD report on pension funds: “In most countries, bonds and equities are the two main asset classes in which pension assets were invested at the end of 2018, accounting for more than half of all investments in 32 out of 36 OECD countries, and 39 out of 46 other reporting jurisdictions” OECD (2019a), *Pension Markets in Focus,* p.29.

6 As noted by van der Zwan (2014), the growing scale disparity between the financial and real sectors has led some authors to assert that the former has in effect become “an increasingly autonomous realm”.

7 This line of thought is exemplified in Mario Seccareccia’s 2012 paper where he sought to adapt monetary circuit theory to the new era of financialisation. Noting the huge growth in financial market scale, and finding that this scale could in no way be reconciled with the monetary needs of productive corporate investment, he concluded that: “Owing to the corporate sector’s position as net lender, rentier speculative behaviour (that Keynes had so vehemently criticised in *The General Theory*) has slowly prevailed in the financial sector and has probably been the largest impetus in pushing this financialisation frenzy into hyper drive over the last decade” (Seccareccia 2012, p. 186). The idea that rentier speculative behaviour is now a major driver of financial scale growth obviously leads to the idea that this development is not ultimately sustainable and will be reversed at some point in the event of a major crisis. Thus, for example, Lavoie argued in a paper published in 2012 that: “Just as the Great Depression called an end to finance capitalism, the current financial crisis should bring about the end of financialisation” (Lavoie 2012, p.232). As concerns the scale dimension of financialisation, this prediction has turned out to be completely wrong. On the contrary, the financial crisis of 2007–2008 gave further impetus to bond market growth as has, to an even greater extent, the current covid crisis.

8 The predominance of FPI flows over FDI flows is clearly indicted by the breakdown of the $6.6 trillion FX daily turnover by counterparty in 2019. Thus, of the $1987 trillion spot turnover, the inter-bank share was 29.8%, as compared with the bank-other financial institutions’ share of 62.2% and the bank-non financial corporation share of 8%.; of the $999 trillion outright forward turnover, the inter-bank share was 26.8% as compared with the bank-other financial institutions’ share of 61.6% and the bank-non financial corporation share of 11.6%; and of the $3202 trillion fx swap turnover, the inter-bank share was 46.8% as compared with the bank-other financial institutions’ share of 48% and the bank-non financial corporation share of just 5%. (BIS 2019).

9 Over the recent period, the excess of foreign holdings of US securities over US holdings of foreign securities has risen at an increasing rate (thus from $2.6 trillion in 2004 ($6.2 trillion versus $3.6 trillion), the excess had risen to $4.8 trillion in 2008 ($8.9 trillion versus $4.1 trillion) and to $7.3 trillion in 2019 ($20.1 trillion versus $12.8 trillion), SIFMA (2020a)

10 As a result of both the rapid increase in borrowing needs and the decline in GDP across OECD economies, the central government marketable debt-to-GDP ratio for the OECD area increased by 13.4 percentage points to around 86% (OECD 2020a).

11 Thus in 2019, central bank holdings of government bonds as percentages of total holdings ranged from 15% for the US to 45% for Japan (OECD 2020a).

12 For further discussion of this point, see Lysandrou (2013).

13 In the first five months of 2020, about 70% of the total government bonds were sold with interest rates below 1% and 27% of the total bonds issued with interest rates between 1 and 2%, and only 5% of total issuance with higher than 2% interest rates (OECD 2020a).
